# Dogs (*Canis familiaris*) Evaluate Humans on the Basis of Direct Experiences Only

**DOI:** 10.1371/journal.pone.0046880

**Published:** 2012-10-08

**Authors:** Marie Nitzschner, Alicia P. Melis, Juliane Kaminski, Michael Tomasello

**Affiliations:** Department of Developmental and Comparative Psychology, Max-Planck Institute for Evolutionary Anthropology, Leipzig, Germany; CNR, Italy

## Abstract

Reputation formation is a key component in the social interactions of many animal species. An evaluation of reputation is drawn from two principal sources: direct experience of an individual and indirect experience from observing that individual interacting with a third party. In the current study we investigated whether dogs use direct and/or indirect experience to choose between two human interactants. In the first experiment, subjects had direct interaction either with a “nice” human (who played with, talked to and stroked the dog) or with an “ignoring” experimenter who ignored the dog completely. Results showed that the dogs stayed longer close to the “nice” human. In a second experiment the dogs observed a “nice” or “ignoring” human interacting with another dog. This indirect experience, however, did not lead to a preference between the two humans. These results suggest that the dogs in our study evaluated humans solely on the basis of direct experience.

## Introduction

Reputation is a key component in human cooperation [1], as well as that of other primate species. For example, chimpanzees use first-hand experience with conspecifics to choose collaborative partners [2]. In contrast, capuchin monkeys failed to use experience of two different human experimenters to choose between them in a token exchange task [3].

There is also evidence in chimpanzees, but not in other great apes, of an ability to form indirect judgments about reputation; that is, assessing others’ behavior as a bystander. Russell et al. [4] found that chimpanzees displayed a preference for a ‘nice’ experimenter after they had witnessed interactions between a beggar and a ‘nice’ person (who gave grapes to the beggar) versus a ‘nasty’ person (who refused to give grapes). However, Subiaul et al. [5] did not find that chimpanzees show a spontaneous preference for a ‘generous’ donor despite following similar methods to those employed in the previous study. Studies with other species suggest that domain-specific skills analogous to reputation judgments are widespread in the animal kingdom [6]–[9]. But these abilities are mostly confined to mating or fighting contexts and are probably highly constrained [5].

As domesticated animals, dogs (*Canis familiaris*) represent an interesting case. Humans actively selected dogs for activities such as hunting, herding, retrieving or guarding [10]. These tasks required intensive social interactions between dogs and humans and therefore a human-driven selection would have favored individuals that were responsive to a broad range of stimuli, such as verbal cues, and had adequate behavioral plasticity, allowing external shaping [11]. For this reason dogs might have evolved some special socio-cognitive abilities, which enabled them to interact and communicate with humans [12]. Several studies suggest that dogs’ cognitive skills in some areas seem to be more flexible than those of species more closely related phylogenetically to humans [13], [14].

In addition dogs use human communicative cues in a highly flexible manner from a very early age and therefore dogs seem to have a high predisposition to develop some understanding of human communication [14], [15]. Finally, dogs’ closest living relatives, the wolves, do not seem to use human given communicative cues as flexibly as dogs even if they are raised under very similar conditions [14], [16], [17]. This seems to change only when wolves are trained in a special way (e.g., by using a clicker) or are exposed extensively to human given communicative cues [18]–[20], but see Gacsi et al. 2009 for an alternative explanation. Taken together these facts suggest that selection processes during domestication affected dogs’ abilities in this domain [17], [21], [22].

Dogs also show other remarkable social capabilities. For example, dogs know when humans’ attentiveness is directed towards them and behave accordingly [23]–[26]. Furthermore they can discriminate between humans using facial cues [27] or scent [28]. Dogs readily form attachments to individual humans [29], and even prefer human company to that of other dogs [30], [31]. These findings suggest that humans are highly relevant social partners for dogs. As this interspecific relationship seems to be crucial for dogs, it would be advantageous for dogs to be able to predict human behavior, based on direct or indirect experience, in order to attach themselves to the more “caring” human.

However, only a few studies have examined dogs’ ability to understand third-party interactions. Two studies provide some evidence that dogs recognize the individual roles of other dogs in a play context with conspecifics [32] and in third-party conflicts [33]. In a study involving interspecific play, Rooney and Bradshaw [34] showed that spectator dogs preferred the winner of a playful tug-of-war game (dog vs. human), irrespective of whether the winner was the dog or the human. But it is important to note that the dogs maintained their preference for the winner even if they had not observed the game beforehand. Thus, it is possible that the dogs were only reacting to subtle signals from the demonstrator dog rather than that they were assessing the human play partner on the basis of the outcome of the game.

Three recent studies are most relevant to dogs’ tendencies to select humans based on their behavior. Petter et al. [35] found that dogs preferred a cooperator over a deceiver. In an object-choice task, subjects approached the cooperative human tester (i.e. who always pointed at the baited container) more often than the deceiving tester (i.e. who always cued the empty container). These results indicate that dogs can learn to differentiate between two strangers based on direct interactions with them. Another recent study hypothesized that dogs are able to make reputation-like inferences after witnessing third-party exchanges between humans. Kundey et al. [36] found that dogs chose the demonstrator who gave food to a human recipient more often than the demonstrator who withheld food. However, the dogs in this study also preferred the human who “gave” food to a wooden box over a human who withheld food, suggesting that rather than forming a reputation based on an observed interaction, dogs simply associated food with one but not with the other experimenter. Marshall-Pescini et al. [37] conducted a similar study and addressed these problems by including a ghost condition, in which no beggar was present. In this control condition, dogs did not prefer one over the other experimenter. This result suggests that dogs did not prefer the generous donor because of her specific behavior, but rather took the beggar-donor interactions into account. However, in this study, a control for potential side preferences was missing, so it is possible that the dogs simply preferred the side from where they saw the food coming and did not use the experimenter-specific information gained through observation of the third-party interactions (see [3] for a similar argument about the behavior of capuchins in a similar setting).

In the current paper we focused on another trait, which might be relevant for dog-human interactions: the human’s attention. The discriminating factor was whether the human paid attention to the dog (nice experimenter) or did not (ignoring experimenter). In the first experiment, we investigated whether dogs can distinguish between a “nice” and an “ignoring” person after having controlled direct interaction with both. In the second experiment, the test dogs only witnessed social interactions as uninvolved bystanders. In these interactions, both experimenters interacted with another dog that was well-known to the subject. Importantly, contrary to Marshall-Pescini et al. [37], our choice situation differed from the experience/demonstration situation in that it required flexibility in their response and ruled out local enhancement as a factor, see [3]. Given the prior studies, which suggest that dogs are able to evaluate humans by eavesdropping, dogs should prefer the “nice” experimenter based on direct experience (experiment 1) as well as on indirect experience (experiment 2).

## Experiment 1

In this experiment we assessed whether dogs use information about the typical behavior of other individuals after they had direct experience with two different female experimenters. The subjects had never interacted with the experimenters previously and only had controlled experiences with both of them during the experience/demonstration phase of this experiment. One of the experimenters engaged in a friendly interaction with the dog, using a cheerful and friendly voice and interacting in a playful manner, whereas the other experimenter ignored the dog and did not speak to him/her. After several interactions with both experimenters subjects were free to choose between the two different experimenters. If they took their experiences into account, i.e. made predictions about the person’s future behavior, they should approach the nice experimenter and/or remain next to that experimenter for a longer period of time given the choice.

### Methods

The procedure of the current study was non-invasive. In Germany, no special permissions for use of dogs in this kind of socio-cognitive studies is required, an IRB approval was not necessary. The two studies were performed in full accordance with German legal regulations and the guidelines for the treatments of animals in behavioral research and teaching of the Association for the study of Animal Behavior (ASAB). All dogs were registered in the dog database of the Department of Developmental and Comparative Psychology (MPI EVA) and were recruited by phone. All dog owners with their dogs participated on a volunteer basis.

### Subjects

Thirty-two dogs, 16 males and 16 females, living as pets with their owners participated in this experiment. Five additional dogs had to be excluded due to being uncomfortable in the testing situation. For more detailed information about subjects in experiment 1, see Table S1 (supplemental material). Only dogs older than one year (mean age +/− SD = 5+/−2.6 years), unfamiliar with both experimenters, and motivated to interact with strangers (according to the owners’ information), were selected from a database of owners who had volunteered to participate in this type of behavioral study. No breed was excluded. The experiment was conducted in a room dedicated to dog studies and the owners were not present during testing.

### Experimental Set-up and General Procedure

The experiment took place in a small empty room (8.70×4 m). The two female experimenters (MN and BM) resembled each other in physical appearance to exclude possible preferences for one or the other physical aspect, but differed in other aspects (clothing, glasses, hairstyle). The dog and the human experimenter interacted directly.

The procedure began with four experience trials per experimenter followed immediately by the first experimental trial. After a break of about 10 minutes the dog received one additional experience trial per experimenter followed by a second experimental trial. This was immediately followed by two additional blocks of one experience trial and one experimental trial, conducted without a break. Every dog participated in seven experience trials with each experimenter and four experimental trials altogether.

### Experience Trials

An unfamiliar helper led the subject on a leash into the testing room and walked around in order to familiarize the dog with the room. Then the helper released the dog from the leash and left the room. After a few seconds the first experience trial started. One experimenter entered the room and interacted with the dog according to her role. The “nice” experimenter behaved in a friendly way, i.e. she displayed play signals such as patting the floor, clapping, petting the dog, friendly shoving the dog and encouraging vocalization in order to establish a playful situation [38, see video in supplemental material]. Importantly, all dogs joined the interactions voluntarily. For more details on the proportions of interactions, see “results” section. The “ignoring” experimenter ignored the dog, i.e. she walked through the room without talking to the dog or making eye contact with the dog. She passed the dog several times, but never reacted to the dog (see video in the supplementary material). Half of the dogs experienced MN being “nice” and half of the dogs experienced MN being “ignoring” (mirrored by the second experimenter BM). Which type of experimenter (nice/ignoring) the dogs experienced first was counterbalanced across subjects and the sequence of experience trials was semi-randomized with no more than two demonstrations by the same experimenter given in a row. The second experimenter entered the room immediately after the first experimenter had left. Each experience trial lasted 30 seconds and there was never more than one experimenter in the room interacting with the subject at a time.

### Experimental Trial

**Figure 1:**
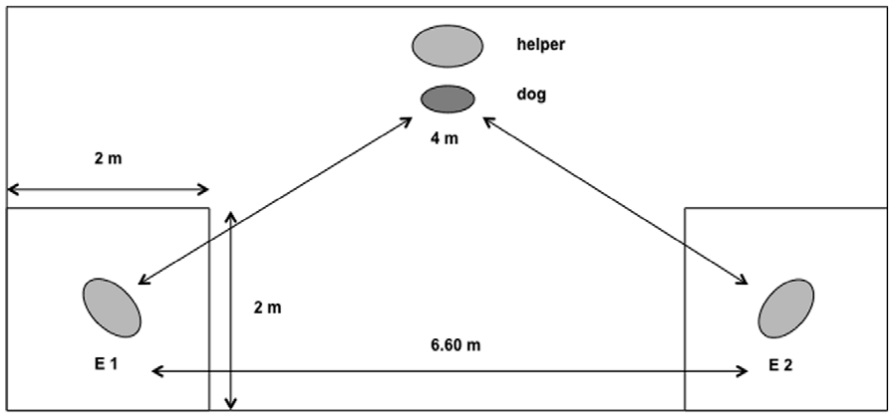
Set up in the experimental trials.

The experimental trials took place in the same room as the demonstrations. Both experimenters entered the room and sat down on the floor, 6.60 m from each other at predetermined locations. The experimenters were seated in different corners of the room with their bodies oriented towards the door from which the dog entered. A 2×2 m area around each experimenter was marked to ensure that the dogs’ approaches towards the human could be coded. When both experimenters were seated at their respective locations, the helper entered the room with the dog on a leash and placed him/her at a predetermined location by the door, equidistant to both experimenters (4 m) (see Fig. 1). After a few seconds the helper released the dog, so the dog could move freely about within the room. During the entire duration of the trial both experimenters remained in the same position with a neutral facial expression and never reacted to or interacted with the dog. One trial lasted 30 seconds. The position of the experimenters was counterbalanced within and across subjects.

### Coding and Analysis

All demonstrations and trials were filmed with four fixed wide-angle cameras and recorded on a Sony DV-Walkman outside the test room. Two cameras filmed the whole testing area from two different corners. The other two cameras observed each 2×2 m area above where the experimenters were sitting during the experimental trials. With this top view we could assess exactly when the subject was within 2 m of the experimenter. During the “nice” experience trials, we coded (1) the duration and the kind of interaction with the experimenter. Interaction was subdivided into cuddling, i.e. the experimenter stroked the dog and talked to him/her in a whisper, and romping, i.e. patting the floor, clapping, shoving and chasing the dog or running away and stimulating the dog with encouraging vocalizations. In addition, we coded (2) exploring, i.e. the dogs moved around the room and were not in the vicinity of the experimenter or were not interested in her, and (3) being stationary, i.e. dogs stayed/sat/laid still. During the “ignoring” experience trials, we additionally analyzed (4) the following behavior, i.e. the dog followed the ignoring experimenter at a short distance or directly beside her, including when the dog became intrusive (e.g. jumping up onto the experimenter), and (5) looking behavior, involving gaze being directed at the experimenter while remaining stationary.

During the experimental trials we coded two measurements: the dog’s first choice between both experimenters, and the duration that the dog stayed in the proximity of the experimenter. First choice was defined as the experimenter that the dog approached first, having at least one paw inside the area that had been marked with tape around the experimenter. We also analyzed the latency for first choice (from the moment the dog was released up until the time that s/he made her first choice; if the dog chose the experimenter in a later than the first trial, 30 s of previous trials without choosing this experimenter were added to the latency). Duration was coded as the amount of time within each trial that the subject stayed next to each experimenter. Again, proximity to the experimenter was coded when at least one of the dogs’ paws was inside the area marked with tape. Additionally, we coded the durations of behaviors of all subjects during the experimental trials. We looked at the exact behavior the dogs showed in proximity to each experimenter: (1) interaction with the experimenter was defined as direct body contact with one of the experimenters, (2) being stationary was defined as the dog standing/sitting/lying next to one of the experimenters, (3) other behavior was coded when dogs spent time inside the taped area, but were engaged in other actions (e.g. sniffing the ground). In addition, the actions outside the taped areas were coded as follows: (1) interaction with the helper, (2) being stationary and (3) other behavior. Experimenter 1 (MN) coded all material from videotape.

A second coder, unaware of the purpose of the study and blind to which experimental condition the dog was in, coded 20% of the video material for reliability purposes. Reliability agreement was excellent for all measures (Choices: Cohen’s Kappa = 1.00, *N_trial_* = 28, *P*<0.001; Duration nice experimenter: Spearman correlation *r* = 0.996, *N_trial_* = 28, *P*<0.001; Duration ignoring experimenter: Spearman correlation *r* = 0.996, *N_trial_* = 28, *P*<0.001). For the behavior analyses of the experience trials, a second coder coded 20% of the experience trials. Reliability agreement reached a high level for all measured durations (Spearman correlations; Cuddling, romping, being stationary, following, looking: all *r* >0.9, *N_trial_* = 98, *P*<0.001; Exploring: *r* = 0.877, *N_trial_* = 98, *P*<0.001). The reliability data for dogs’ behavior in experimental trials reached an excellent level of agreement in the following measures: interaction nice, being stationary nice, other behavior nice, interaction ignoring, being stationary ignoring, other behavior ignoring, being stationary outside, other behavior outside: all *r* >0.9, *N_trial_* = 28, *P*<0.001. Reliability agreement for the interaction with the helper did not reach an acceptable level *r* = 0.39, *N_trial_* = 28, *P* = 0.036), which is why this measure was not used for further analysis.

All analyses were done using SPSS 16. Trials in which the dogs chose none of the experimenters were excluded from the analysis (28.1%). The number of dogs that chose at least one experimenter dropped over trials (*N_Trial1_* = 31, *N_Trial2_* = 27, *N_Trial3_* = 15, *N_Trial4_* = 19). We checked whether assumptions for parametric tests were fulfilled by applying Levene’s Test and by visually inspecting plots of residuals versus expected values. Both indications showed violations of the assumptions (duration nice experimenter: *F*_3,28_ = 3.475, *P* = 0.029; duration ignoring experimenter *F*_3,28_ = 3.039, *P* = 0.045). Based on theses results we used non-parametric exact test statistics. We used Wilcoxon exact signed-ranks test for analyses within groups and Mann-Whitney U test for analyses between groups. All statistical tests were two-tailed and the alpha level was set to 0.05.

### Results

During the “nice” experience trials, the dogs spent 88.9% of each trial interacting with the experimenter (range: 60.4%–100%). In 61.9% of interaction time the experimenter cuddled the subject and in the remaining interaction time (38.1%) she played with the dog. The subjects explored the room over 5.6% of the trial time and were stationary 4.9% of the trial time. For the “ignoring” trials the proportion was as follows: Exploration 35.7%, following behavior 20.4%, being stationary 42.8%. While the dogs were stationary, they gazed at the experimenter 58.6% of the time.

First experimental trial analysis showed that the dogs did not approach the nice experimenter or the ignoring experimenter significantly more often (*N_nice_ = *19, *N_ign_ = *13, Binomial test: *P* = 0.377). Furthermore, in the first trial subjects did not spend more time next to one or the other experimenter (mean nice: 6.9 s; mean ignoring: 5.2 s; Wilcoxon exact signed-ranks test: *T* = 281.00, *N* = 30 (2 ties), *P* = 0.325). If we compare the median percentage of first approaches across all experimental trials, subjects did not prefer to approach one of the experimenters more often (nice E: 67%, ignoring E: 33% of trials, Wilcoxon exact signed-ranks test: *T* = 204.50, *N* = 25 (7 ties), *P* = 0.252). Analysis of latencies revealed that the dogs did not approach one experimenter faster than the other (mean nice: 12 s; mean ignoring: 26 s; *T* = 177.5, *N* = 32, *P* = 0.107).

**Figure 2:**
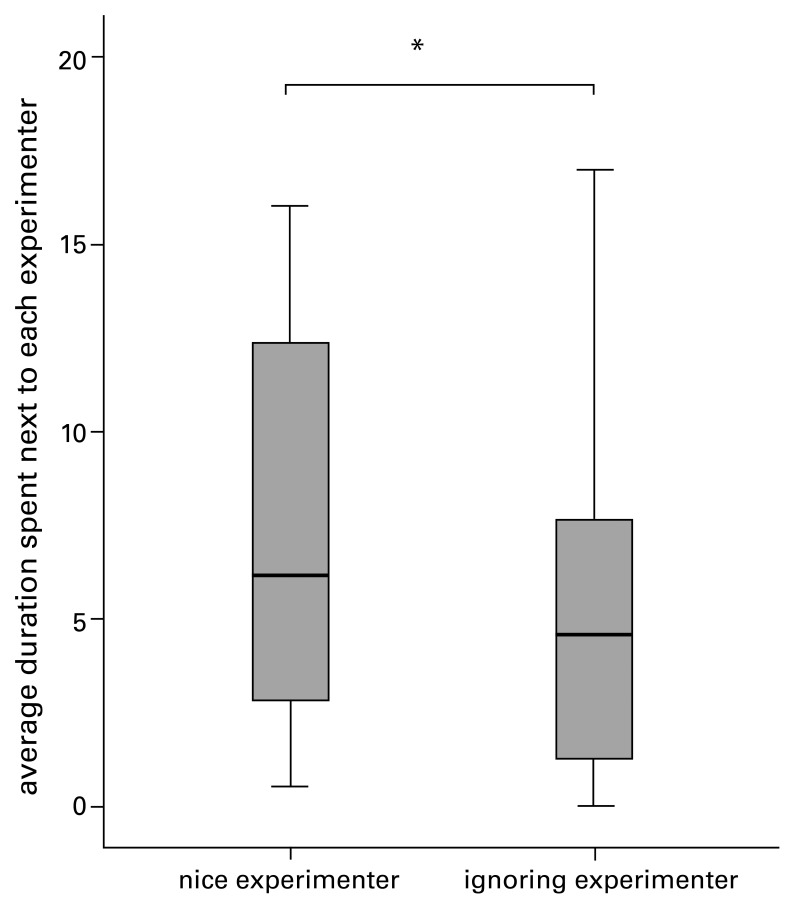
Average duration spent next to each experimenter in experiment 1. Figuer 2 displays medians and interquartiles of the average duration the dogs stayed in the proximity of an experimenter over all trials (in experiment 1).

However, comparing the median time spent in proximity to each experimenter over all trials, the dogs stayed close to the nice experimenter longer than they did to the ignoring experimenter (*T* = 336.5, *N* = 30 (2 ties), *P* = 0.031) (see Fig. 2). There was also a correlation between an individual’s time spent next to the nice experimenter over all trials and an individual’s percentage of first approaches to the nice experimenter (*r* = 0.388, *N* = 32, *P* = 0.028).

A comparison between females and males with regard to the time spent in proximity to each experimenter over all trials revealed no significant differences (Mann-Whitney U test: duration nice experimenter: *U* = 105.0, *N_f_* = 16, *N_m_* = 16, *P = *0.396; duration ignoring experimenter: *U* = 105.0, *N_f_* = 16, *N_m_ = *16, *P = *0.396).

Analyses of the durations close to the nice vs. ignoring experimenter for the first and the second half of trials separately revealed a difference in the first half of trials (trial 1 and trial 2; mean duration spent close to nice E: 7.3 s, mean duration spent close to ignoring E: 5.0 s; *T* = 346.5, *N* = 31 (1 tie), *P* = 0.05) but not in the second half (trial 3 and trial 4; mean time spent close to nice E: 8.2 s, mean time spent close to ignoring E: 5.9 s: *T* = 151.0, *N* = 21, *P* = 0.23).

We also tested whether dogs preferred one of the two female experimenters (MN or BM). We found no preference in the mean duration over all trials (*T* = 205.50, *N* = 30 (2 ties), *P* = 0.59) as well as in the duration of the first trial (*T* = 288.00, *N* = 30 (2 ties), *P* = 0.26).

Given the difference in durations close to the nice vs. the ignoring experimenter, we also coded subjects’ more detailed behavior towards the two experimenters. The more detailed behavior of the subjects during all experimental trials is composed as follows. Behavior in close vicinity to the experimenters (inside the tape marked areas): interaction with: nice 6.3% vs. ignoring 6.5% (*T* = 253.5, *N* = 31 (1 tie), *P* = 0.919); being stationary: nice 5.3% vs. ignoring 2.9% (*T* = 73.0, *N* = 14 (18 ties), *P* = 0.217); other behavior: nice 10.1% vs. ignoring 6.9% (*T* = 382.0, *N* = 32, *P* = 0.027). In the remaining experimental trial time (i.e. outside the tape marked areas) subjects were stationary 27.4% of the time; they interacted with the helper 3.5% of the time and engaged in other behavior 30.1% of the time. There was no detectable difference in the detailed behavior towards the two experimenters in trial 1 (Wilcoxon exact signed-ranks test: all *P*>0.2).

### Discussion

In this experiment we found that dogs stayed next to the “nice” experimenter longer than they did to the “ignoring” experimenter. This indicates that the dogs used their knowledge about the experimenter’s behavior based on the direct experiences that they had had with them. Local enhancement and location relative to the experimenters [3] can be both excluded as determining factors in the dogs’ choice because the experience trials did not occur in a particular location within the testing room and were therefore disassociated from the experimenters’ subsequent location within the room during the experimental trial.

An analysis of the first and the second half of trials separately revealed a preference for the nice experimenter only in the first half of trials. The fact that they did not prefer the nice experimenter in the second half of trials could be explained by a fatigue effect, which might confound the results of later trials. The subjects may have lost motivation because both experimenters did not react to them during the experimental trials, although their roles were enforced during the course of the experience trials. Another explanation for the absence of significant differences in the second half of trials could simply be the smaller sample size, since almost one third of the subjects stopped choosing either experimenter (descriptive data support this possibility). Again, this is not surprising since in the first two experimental trials dogs had direct experience with both experimenters and had the opportunity to learn that none of them would interact with them in the experimental trials. Interestingly the dogs did not show any preference in the very first trial irrespective of the measure used. It could be that the dogs had to get used to the different setup of the experimental trials compared to the experience trials (i.e. experimenters were sitting still instead of walking around the room).

We found no preference for the nice experimenter in the subjects’ first choices. One explanation could be that the sample size for that analysis was too small as the percentage of the “nice” choices were in fact correlated with the time spent next to the nice experimenter. Another possible explanation for this result is that approaching is simply not a costly behavior for dogs. In contrast, spending time with the human may be more important, which is why dogs showed a preference in the amount of time they spent with one over the other experimenter.

## Experiment 2

In this experiment we tested whether the dogs developed a preference for a nice experimenter after having had indirect experiences with her. As in the first experiment, subjects were not familiar with any of the experimenters and only had controlled experiences with them. In this experiment subjects did not interact directly with the humans, but instead observed interactions between a “nice” experimenter and a demonstrator dog, and an “ignoring” experimenter and a demonstrator dog. The prediction was that if dogs are able to form reputation judgments based on third-party interactions, they should preferentially approach the nice experimenter first and/or stay next to her for longer.

### Methods

#### Subjects

Thirty-two dog pairs participated in this experiment. All dogs lived as pets with their owners. From sixty-four dogs 32 served as subjects (16 males, 16 females), and the other 32 dogs participated as demonstrator dogs. Four additional dog pairs had to be excluded for several reasons (one subject never chose any of the experimenters, one subject constantly jumped over the Plexiglas barrier during demonstrations, two demonstrator dogs were not motivated to interact with the nice experimenter). Each of twenty-nine dog pairs lived together in one household. The three remaining dog pairs knew each other well. None of the dogs participated in experiment 1. As in the previous experiment only dogs older than one year (mean age +/− SD = 5.4+/−3.4 years), unfamiliar with both experimenters and motivated to interact with strangers (according to the owners’ information) were selected from a database of volunteer owners. For information about the subjects’ name, breed, sex, age on the test day and social rank related to the partner dog see Table S1 (supplemental material). No breed was excluded. The owners were not present during testing.

### Experimental Set-up and General Procedure

**Figure 3:**
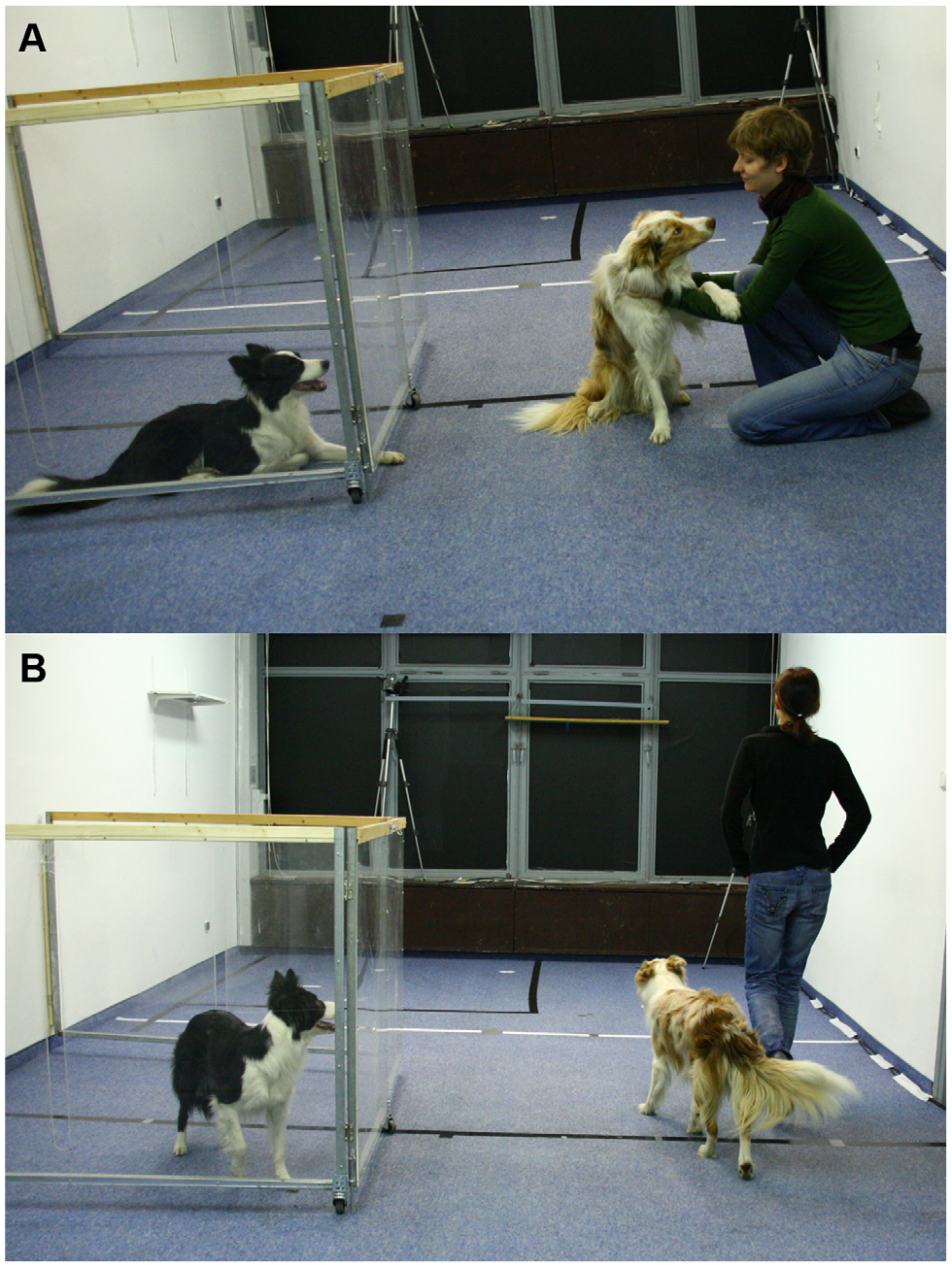
Demonstration in experiment 2 (A nice experimenter, B ignoring experimenter).

The experiment took place in the same room as experiment 1. The two experimenters were the same as in the previous experiment (MN and BM), but here they did not interact directly with the subjects. Instead, the subject stayed behind a foldable Plexiglas partition (about 1.80×1.70 m) (see Fig. 3) in the middle of the test room and observed the experimenters interacting with their partner dog.

The procedure began with four demonstration trials per experimenter followed immediately by the first experimental trial. After this first block there was a break of about 10 minutes. Subsequently, the dog received one additional demonstration trial per experimenter followed by a second experimental trial. This was immediately followed by two additional blocks of one experience trial and one experimental trial, conducted without a break. So that every dog received seven demonstrations per experimenter and four experimental trials altogether. Which role (ignoring vs. nice) each experimenter (MN and BM) played was counterbalanced across subjects.

### Demonstration Trials

An unfamiliar helper led the subject on a leash into the testing room and walked around in order to familiarize the dog with the room. Afterwards s/he positioned the dog behind the Plexiglas partition (see Fig. 3). Then, the partner dog entered the testing room and after a few seconds the first demonstration began. The experimenters behaved in the same way as in experiment 1. The “ nice” experimenter behaved in a friendly manner towards the demonstrator dog and played with her while the “ignoring” experimenter ignored the demonstrator dog. Both experimenters paid no attention to the subject behind the Plexiglas partition. Before each experimental trial the helper led the partner dog into another room and afterwards led the subject out of the testing room. Again, the sequence of the demonstrations was semi-randomized (no more than two demonstrations by the same experimenter were given in a row) and the type of experimenter that the dogs experienced first was counterbalanced across subjects. Each demonstration lasted 30 seconds and there was only ever one experimenter in the room at a time with the demonstrator dog.

### Experimental Trial

After both dogs had left the testing room together with the helper, the two experimenters entered the testing room and folded the Plexiglas partition so that it was flat against the wall. They sat down on the floor, 6.60 m from each other (so that the set up and the procedure were the same as in the experimental trials of experiment 1). The bodies of the experimenters were oriented towards the door from which the dog entered. Again, a 2×2 area was marked with tape around each experimenter ensuring that dogs’ approaches could be coded. The helper entered the room with the dog on a leash and placed him/her equidistant to both experimenters (4 m). Each experimental trial lasted 30 seconds. During this time the experimenters remained in their positions with neutral facial expressions and refrained from reacting to the dog’s behavior. The positions of the experimenters (nice and ignoring) were counterbalanced within and across subjects.

### Coding and Analysis

This experiment was recorded in the same way as experiment 1. During the demonstration trials, we coded the same behaviors as in experiment 1 for the demonstrator dog. Additionally, the behavior of the observer dog was also analyzed. We coded (1) the looking time towards the experimenter, (2) the time that the dogs stayed/laid/sat still, (3) the time subjects vocalized (including all vocalizations such as barking, whining and whimpering) and (4) the time subjects spent scratching or jumping up onto the barrier.

During experimental trials, again, the dependent measures were first choice, i.e. the experimenter (ignoring or nice) that the dogs chose to approach first, and duration spent in the vicinity of each experimenter, i.e. how long the subject stayed close to each experimenter. Furthermore, we computed the latencies for first choices. For all measures we used the taped area around the experimenters (2 m) to operationalize whether the subject was close to the experimenter or not. Again, we coded the exact behavior of subjects during the experimental trials with the same definitions as in experiment 1: (1) interaction with the experimenter, (2) being stationary, (3) other behavior. Actions outside the taped areas were coded with the same criteria: (1) interaction with the helper, (2) being stationary and (3) other behavior. All trials were coded from video by experimenter 1 (MN). For reliability purposes a second coder (the same as in experiment 1; unaware of the purpose of the study and blind to the experimental condition) coded 20% of the video material. The agreement for the first choice data reached 100% (Cohen’s Kappa = 1.0, *N_trial_* = 26, *P*<0.0001). The reliability agreement for the time spent near the experimenters was excellent (Duration nice experimenter: Spearman correlation *r* = 0.995, *N_trial_* = 26 *P*<0.001; Duration ignoring experimenter: *r* = 0.989, *N_trial_* = 26, *P*<0.001). For the behavior analyses, a second coder coded 20% of the demonstration trials. Reliability agreement for the demonstrators’ behavior reached high levels for all measurements (Spearman correlations; Cuddling, romping, exploring, being stationary, following: all *r* >0.9, *N_trial_* = 98, *P*<0.001; Looking: *r* = 0.879, *N_trial_* = 98, *P*<0.001). Reliability analyses for subjects’ behavior reached high agreement in most measures (Vocalization: *r* = 0.900, *N_trial_* = 98, *P*<0.001; Jumping and scratching on barrier: *r* = 0.904, *N_trial_* = 98, *P*<0.001; Being stationary: *r* = 0.865, *N_trial_* = 98, *P*<0.001) and an acceptable level of agreement for looking at the experimenter (*r* = 0.721, *N_trial_* = 98, *P*<0.001). The reliability agreement for the dogs’ behavior in experimental trials was excellent for all measurements associated with both experimenters: interaction nice/ignoring, being stationary nice/ignoring, other behavior nice/ignoring (all *r* >0.9, *N_trial_* = 28, *P*<0.001), for being stationary (*r* = 0.957, *N_trial_* = 28, *P*<0.001) and for other behavior outside the taped areas (*r* = 0.971, *N_trial_* = 28, *P*<0.001). Level of agreement for helper interactions reached an acceptable level: *r* = 0.753, *N_trial_* = 28, *P*<0.001.

All analyses were done on SPSS 16. Again, all trials in which none of the experimenters were chosen were excluded from the analysis (23.6%). As in experiment 1, the number of dogs that chose at least one experimenter dropped over trials (*N_1_* = 30, *N_2_* = 23, *N_3_* = 21, *N_4_* = 20). We aborted two tests after the first experimental trial because the demonstrator dogs were no longer motivated to interact with the “nice” experimenter. The first trial data of both dogs were included in analysis. For a better comparability of the data with those of experiment 1, we used non-parametric exact test statistics. We used Wilcoxon exact signed-ranks test for analyses within groups and Mann-Whitney U test for analyses between groups. All statistical tests were two-tailed and the alpha level was set to 0.05.

### Results

Demonstrator dogs interacted 92.8% of trial time with the “nice” experimenter (range: 79.8%–99.8%). In 59.7% of the interaction time, the experimenter cuddled the demonstrator and in the remaining interaction time (40.3%), she played around with the dog. During the “nice” trials the dogs spent a marginal proportion of the time exploring the room (5.5%) and being stationary (1.4%). During the “ignoring” trials, demonstrators spent 43.3% of the time exploring; they followed the “ignoring” experimenter 31.6% of the time and were stationary in 24.6% of the trial time. While dogs were stationary they gazed at the experimenter 64.1% of the time. Analyses of subjects’ behavior revealed significant differences in “nice” demonstrations versus “ignoring” demonstrations in certain aspects. Dogs vocalized more during “nice” demonstrations than during “ignoring” demonstrations (mean nice: 27.0% vs. mean ignoring: 12.5%, Wilcoxon exact signed-ranks test *T* = 23.0, *N* = 26 (6 ties), *P*<0.001), they spent more time scratching and jumping up on the barrier in the “nice” trials than in the “ignoring” trials (mean nice: 5.3% vs. ignoring: 3.2%, *T* = 52.0, *N* = 21 (11 ties), *P* = 0.03) and they looked longer towards the experimenter (mean nice: 80.4% vs. ignoring 74.9%, *T* = 142.0, *N* = 32, *P* = 0.02). In addition, subjects tended to sit, stay or lay still longer during the “ignoring” demonstrations (mean nice: 73.2% vs. ignoring: 78.6%, *T* = 356.0, *N* = 32, *P* = 0.085).

Regarding data analyses of experimental trials, subjects did not show any preference for approaching the nice experimenter first in the first trial (*N_nice_ = *19, *N_ign_ = *13, Binomial: *P* = 0.377). Also if we compare the median percentages of first approaches over all trials, we found no preference for the nice experimenter or the ignoring experimenter (*T* = 146.50, *N* = 21 (11 ties), *P* = 0.284). We also found no differences in the latencies to approach one or the other experimenter (mean nice: 12 s; mean ignoring: 19 s; *T* = 259.5, *N* = 32, *P* = 0.938).

**Figure 4:**
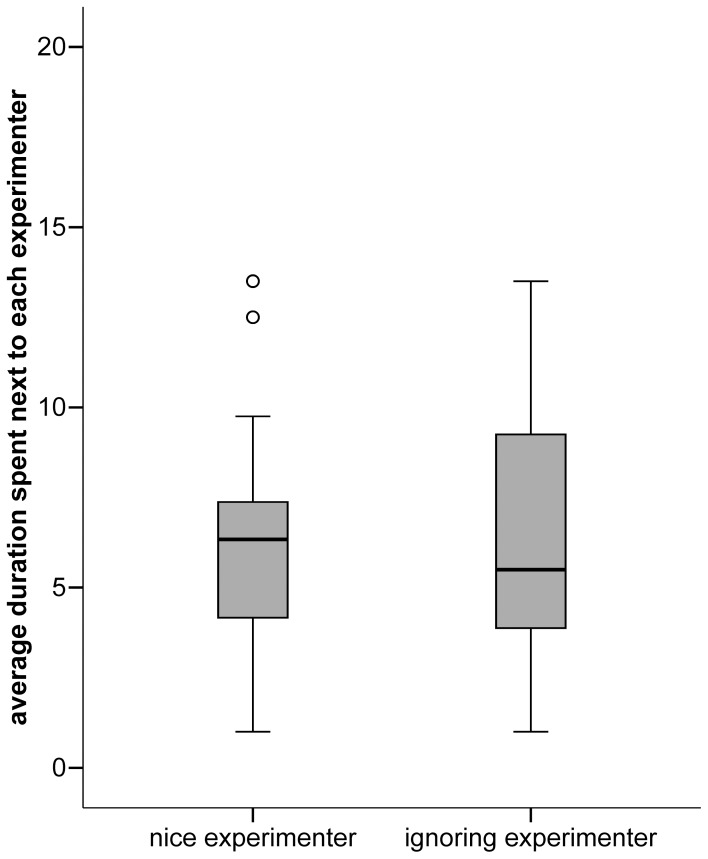
Average duration spent next to each experimenter in experiment 2. Figure 4 displays medians, interquartiles and outliers of the average duration the dogs stayed in the proximity of an experimenter over all trials (in experiment 2).

Subjects in this experiment did not stay close to the nice experimenter longer than to the ignoring experimenter in the first trial (mean nice: 6.5 s; mean ignoring: 6.8 s; Wilcoxon exact signed-ranks test: *T* = 211.50, *N* = 29 (3 ties), *P* = 0.902) or over all trials (*T = *215.5, *N* = 29 (3 ties), *P* = 0.970) (see Fig. 4). When we compare results split by sex, we found no differences within groups as well as between groups in all trials (all *P*>0.1) and in the first trial (all *P*>0.3).

We found no preference for the nice or the ignoring experimenter analyzing the first and the second half of trials separately. Subjects did not stay next to any experimenter for longer in the first half (nice vs. ignoring *T* = 255.5, *N* = 30 *P = *0.644) or in the second half (nice vs. ignoring *T* = 83.5, *N* = 23, *P = *0.168).

Comparing the mean duration over all trials, dogs tended to stay in proximity to BM for longer (*T* = 132.00, *N* = 29 (3 ties), *P* = 0.064). However, they did not prefer to stay close to one of the female experimenters in the first trial (*T* = 214.5, *N* = 29 (3 ties), *P* = 0.953).

We coded the detailed behavior of the subjects during the experimental trials. We found no difference in the dogs’ behaviors towards the nice or ignoring experimenter over all trials: interaction (nice, mean percentage: 4.5% vs. ignoring, mean percentage: 4.6%; *T* = 223, *N = *28 (4 ties), *P* = 0.662), being stationary (nice 1.4% vs. ignoring 1.4%, *T* = 23, *N* = 9 (23 ties), *P* = 1.00), other behavior (nice 14.0% vs. ignoring 14.4%, *T* = 259, *N* = 32, *P* = 0.934). During the remaining experimental trial time (outside the taped area), dogs’ behavior was composed as follows: being stationary (11.7%), interaction with the helper (1.8%), other behavior (45.6%). There was no detectable difference in the detailed behavior towards the two experimenters in trial 1 (all *P*>0.4).

### Discussion

Interestingly, in this experiment we found that subjects behaved differently during demonstration trials depending on which demonstration (nice vs. ignoring) was performed. This suggests that subjects were attentive and distinguished the different types of dog-human interactions. In spite of this fact, we found that dogs had no preference for any type of experimenter. The dogs did not prefer to stay longer next to the nice experimenter nor did they choose the nice experimenter more often in their first approach. Furthermore, subjects did not prefer the nice experimenter in the first trial and in the first half of trials, suggesting that there was no preference even without the possible confounds of contradictory information during the experience and the experimental trials. These results indicate that the dogs in our setup did not use the information gained through witnessing third party interactions. Besides, we found no evidence that the factor sex had any effect on the results of this experiment. Instead, we found that the dogs tended to stay close to one of the female experimenters (BM) for longer. This finding may indicate that dogs are not interested in the experimenter’s relationship with another dog but simply assess the experimenter’s disposition through the help of traits which the experimenter portrays (e.g. smell, dominance appearance).

## General Discussion

The results of the current study suggest that dogs take direct experience into account when they have to choose between two different experimenters. However, we did not find any evidence supporting the hypothesis that they use indirect acquired information flexibly, at least in situations in which no food is involved and subjects observe a human interacting with another dog.

The first experiment showed that dogs preferred to stay next to the experimenter who behaved in a friendly manner towards them rather than to the experimenter who ignored them. This is an indication that dogs did rely on the direct experience and paid attention to the experimenters’ roles.

The results of the second experiment suggest that dogs do not form indirect reputation judgments after observing interactions between familiar dogs with two different unfamiliar experimenters (nice vs. ignoring; same as in experiment 1). They did not show any preference for one or the other experimenter role. Instead we found a trend in preference for one of the female experimenters (BM), although we tried to control for that (both experimenters resembled each other in appearance to exclude preferences for physical aspects). Apparently, dogs in our study did not use the less accurate information about the experimenters’ roles. These findings are not consistent with the results of Kundey et al. [36] and Marshall-Pescini et al. [37], where dogs supposedly made reputation-like inferences. One possible explanation might be that the recipient in our study was a conspecific instead of a human and could therefore be argued that the demonstrated interactions were less relevant for the subject than the human-human interactions in the previous studies. This assumption would be supported by a few studies on dogs’ attachment to humans, which have shown that dogs prefer to stay with a human being than with a conspecific [30], [31]. However, since in most instances our subjects in experiment 2 live with conspecifics in one household and/or go to a dog day care regularly and therefore witness dog-human-interactions frequently, we assume that they are probably as good at evaluating a human engaging in interactions with another dog as with another human [39]. On the other hand, having permanent contact with the partner dog could have also led to a rather competitive than affiliative relationship. We assume that dog pairs in one household typically have an affiliative relationship but we cannot totally rule out that competitive tendencies in their relationship also had an influence on the results.

We think one important factor that differentiates this study from previous ones is the exclusion of food from our study. We are aware of the high motivating effect of food, but we encountered several problems with the use of food in this kind of setup in an extensive pilot study (Nitzschner et al., unpublished data). In this pilot phase, we found that the dogs did not develop a preference for the ‘giving donor’, even after many direct experiences. A possible explanation for this could be that the dogs focused their attention on the food more than on the behavior of the experimenters. The results of Kundey et al. [36] have shown that dogs choose a giving experimenter even if social partners were replaced by non-living, and thus non-social, objects (i.e. a small moving box as a recipient). This fact provides some evidence that dogs merely reacted to the demonstrators’ behavior in relation to the food instead of the actual social interactions. In Marshall-Pescini et al. [37], the demonstrators did not change positions between the demonstrations and test situation. Therefore, an alternative explanation is that the dogs only preferred the side where they saw the food exchange or where they preferentially looked during the observation phase, instead of taking the humans’ interaction into account and being able to keep track of “which” human was the donor. This is a very important difference from our study. Here the setup in the experience/demonstration trials differed from the setup in the experimental trial. Therefore we can rule out local enhancement.

Another possibility for the absence of preferences in experiment 2 could be that dogs could not distinguish between the experimenters due to the fact that they had no direct contact with them. But this hypothesis is relatively unlikely as dogs are known to have no problems distinguishing between different people based on facial cues [27] or by scent alone [28]. Both traits as well as other characteristics (hairstyle, clothes, glasses etc.) were available throughout the procedure (i.e. through a transparent Plexiglas barrier and through slots in the barrier). Besides, the dogs tended to prefer one of the female experimenters (BM), which indicate that they were able to distinguish between both humans.

Another potential pitfall in experiment 2 could be that subjects perceived the “ignoring” experimenter as a human who went for a walk with the demonstrator dog. In fact, the demonstrators displayed following behavior in approximately one third of the “ignoring” demonstration time. We cannot totally exclude this possibility, but in our opinion it is unlikely, because the rest of the time the demonstrator dogs did not stay in the proximity of the “ignoring” experimenter (in contrast to the “nice” demonstrations in which the demonstrators interacted with the experimenter roughly 92.8% of the time). Additionally the “ignoring” experimenter never talked to the demonstrator dog (as opposed to the “nice” one). Voice is considered to be a very salient cue [37], [39], which should clearly demonstrate a difference in the human’s attentional state towards the demonstrator dog.

However, our study provides some hints that dogs apply their knowledge about the behavior of two different strangers gained through direct interactions, but that they do not use the information about humans’ behavior when they have witnessed social interactions between the human actors and another dog in order to choose their social partners. These results cast some doubt on the flexibility of the reputation-like skill previously reported by other studies [36], [37] and suggest potential limitations (at least when no food is involved and dogs observe human-dog interactions) in their ability to extract, or transfer respectively, relevant social information when observing third-party interactions. Given the small number of publications on this topic, future research could investigate the factors constraining dogs’ ability in this domain; for example testing this method with other experimenter roles or presenting dogs with human-human (e.g. experimenter-owner) rather than human-dog interactions.

## Supporting Information

Table S1 Subject list.
(DOCX)
