# Supplementary material for: Dogs (Canis familiaris) Evaluate Humans on the Basis of Direct Experiences Only
Source: PLoS One. 2012 Oct 8;7(10):e46880. doi: 10.1371/journal.pone.0046880 (PMC3466196; doi:10.1371/journal.pone.0046880)
Supplement: Table S1 — Subject list. (DOCX) [file pone.0046880.s001.docx]

Table S1: Name, sex, breed and age of subjects (in years) in experiment 1 (I) and experiment 2 (II). * = spayed/neutered

| **Name** | **Sex** | **Breed** | **Age** | **Exp.** |
| --- | --- | --- | --- | --- |
| Milka | F | Labrador Retriever | 7 | I |
| Gusti | M | Tibet Terrier | 5 | I |
| Cantor | M | German Shepherd | 5 | I |
| Arthos | M | Labrador Retriever | 9 | I |
| Kira | F | French Bulldog | 2 | I |
| Ginger* | F | Mongrel (Labrador Retriever X Schnauzer) | 3 | I |
| Elliot | M | Mongrel | 2 | I |
| Ned | M | Border Collie | 2 | I |
| Ronja | F | Border Collie | 4 | I |
| Maxi | F | Mongrel (Malinois X Tervueren) | 2 | I |
| Frieda | F | Biewer Terrier | 4 | I |
| Jimmy | M | Labrador Retriever | 9 | I |
| Rex * | M | Mongrel (German Shepherd) | 7 | I |
| Bodo * | M | Mongrel (Malinois X German Shepherd) | 6 | I |
| Pauline | F | Portuguese Water Dog | 9 | I |
| Auguste | F | Mongrel (Border Collie) | 10 | I |
| Brutus | M | Bullterrier | 2 | I |
| Chima* | M | Rhodesian Ridgeback | 8 | I |
| Luna * | F | Bearded Collie | 7 | I |
| Casy * | F | Labrador Retriever | 6 | I |
| Asko | M | Labrador Retriever | 5 | I |
| Melli * | F | Mongrel (Weimaraner X Labrador Retriever) | 6 | I |
| Blanka | F | Labrador Retriever | 5 | I |
| Trulla | F | Border Collie | 6 | I |
| Balou | M | Flat Coated Retriever | 8 | I |
| Pine* | F | German Shorthaired Pointer | 9 | I |
| Romy | F | Mongrel (Rottweiler) | 2 | I |
| Ben | M | German Shepherd | 2 | I |
| Pepe* | M | Weimaraner | 5 | I |
| Feli | M | Jack Russell Terrier | 5 | I |
| Max | M | Swiss Mountain Dog | 3 | I |
| Maylo* | F | Mongrel (Schnauzer) | 11 | I |
| Fight | M | Miniature Dachshund | 4 | II |
| Itchi | F | Shih Tzu | 2 | II |
| Filou* | M | Mongrel (German Spitz) | 3 | II |
| Balu | M | Shih Tzu | 4 | II |
| Gustav* | M | Labrador Retriever | 9 | II |
| Luna 2 | F | Malinois | 8 | II |
| Bolli * | F | Mongrel | 4 | II |
| Luke | M | Mongrel (Rhodesian Ridgeback) | 7 | II |
| Jonas | M | Leonberger | 3 | II |
| Cora * | F | Mongrel (Podenco) | 7 | II |
| Indira* | F | Berner Sennenhund | 5 | II |
| Max* | M | Mongrel | 10 | II |
| Paula | F | Mongrel (Hovawart) | 4 | II |
| Nikolaus | M | Whippet | 9 | II |
| Ocatarian | M | Whippet | 7 | II |
| Ali * | M | Mongrel (Doberman Pinscher) | 7 | II |
| Emily* | F | Mongrel | 12 | II |
| Baerbel | F | Malinois | 4 | II |
| Lotti | F | Golden Retriever | 1 | II |
| Balou * | M | Berger Blanc Suisse | 3 | II |
| Samson | M | Berger Blanc Suisse | 3 | II |
| Freddy* | F | Mongrel (Labrador Retriever) | 5 | II |
| Bella* | F | Mongrel (Yorkshire Terrier X Poodle) | 3 | II |
| Karah* | F | Labrador Retriever | 7 | II |
| Cianna | F | Staffordshire Terrier | 14 | II |
| Fix | M | Malinois | 1 | II |
| Bacardi* | F | Mongrel (German Shepherd) | 9 | II |
| Judy | F | French Bulldog | 1 | II |
| Wuma | F | Beagle | 3 | II |
| Pitscher* | M | Australian Shepherd | 9 | II |
| Booker* | M | Australian Shepherd | 1 | II |
| Jazz | M | Border Collie | 6 | II |
